# New Advances in Anti-HIV-1 Strategies Targeting the Assembly and Stability of Capsid Protein

**DOI:** 10.3390/ijms26125819

**Published:** 2025-06-17

**Authors:** Chengfeng Zhang, Benteng Li, Jiamei Li, Haihong Zhang, Yuqing Wu

**Affiliations:** 1 State Key Laboratory for Supramolecular Structure and Materials, College of Chemistry, Jilin University, No. 2699 Qianjin Street, Changchun 130012, China; 2 Institute of Theoretical Chemistry, College of Chemistry, Jilin University, No. 2 Liutiao Road, Changchun 130023, China; 3 National Engineering Laboratory for AIDS Vaccine, School of Life Science, Jilin University, No. 2699 Qianjin Street, Changchun 130012, China

**Keywords:** HIV-1, capsid protein, small-molecule inhibitors, molecular mechanism, protein–protein interactions (PPIs)

## Abstract

The HIV-1 capsid has emerged as a highly attractive drug target due to its highly conserved sequence and critical role in the viral life cycle. By disrupting interactions between capsid proteins and impairing the proper assembly or disassembly of the capsid, the inhibitors can effectively suppress HIV-1 replication and infection. Based on this mechanism, numerous small-molecule agents targeting the HIV-1 capsid protein have been developed to date. In this review, we report the latest advances in such inhibitors and delve into their molecular mechanisms of action. We find a focus on small molecules modulating capsid stability and their assembly/disassembly. Hopefully this study will further enhance the understanding of HIV-1 inhibition mechanisms, facilitating the future exploration of novel capsid inhibitors.

## 1. Introduction

Acquired immunodeficiency syndrome (AIDS) is a chronic infectious disease caused by the human immunodeficiency virus (HIV). HIV comprises two genetic types: HIV-1 and HIV-2. HIV-1 is the predominant epidemic pathogen, characterized by stronger infectivity and pathogenicity, while HIV-2 exhibits lower pathogenicity and slower progression. By the end of 2023, an estimated 39.9 million people worldwide were living with HIV, underscoring its continued status as a major global public health challenge [1]. According to the latest WHO statistics, approximately 30.7 million HIV-infected individuals globally are receiving antiretroviral therapy (ART) [1]. However, ART requires lifelong adherence and may lead to viral resistance against existing drugs, necessitating the development of inhibitors targeting novel new mechanisms to combat the infection.

The frequent occurrence of viral infections and the rapid evolutionary characteristics of their pathogens pose persistent threats to global public health security. The accumulation of mutations in viral genomes not only drives the continuous emergence of novel infectious diseases but also exacerbates the challenge of drug resistance against existing antiviral therapies [2,3,4]. Consequently, exploring novel antiviral targets and developing broad-spectrum therapeutics with high resistance barriers have become urgent priorities in antiviral research [2,5]. The viral capsid is a highly ordered protein shell that encapsulates the viral genome, formed through the self-assembly of single or multiple types of capsid protein subunits via non-covalent and/or covalent interactions [6,7,8]. As the core structural unit maintaining viral particle integrity, the capsid protein coordinates multiple stages of the viral life cycle: establishing a physical barrier to protect the viral genome from degradation by host nucleases, mediating host cell invasion through surface receptor recognition domains, and participating in the regulation of viral particle assembly and release processes [5,9,10]. Therefore, any abnormalities in the capsid assembly or disassembly processes can significantly impair viral infectivity, making these processes highly attractive targets for drug development [11].

**Figure 1:**
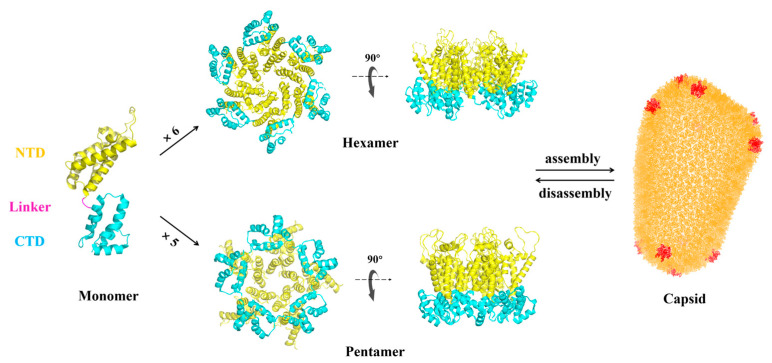
Structures of the HIV-1 CA monomer (PDB: 4XFX), hexamer (PDB: 4U0D), pentamer (PDB: 5MCY), and capsid (PDB ID: 3J3Q). The N-terminal domain (NTD) shown in yellow; flexible linker shown in magenta; C-terminal domain (CTD) shown in cyan. Pentamers are shown in red in the complete capsid lattice (brightorange).

The mature HIV-1 capsid is a fullerene cone-shaped structure (mature core) encapsulating the viral RNA genome, playing critical roles in both early and late stages of viral replication. The HIV-1 capsid protein (CA), also known as p24, is a structural protein derived from the proteolytic cleavage of the Pr55Gag precursor encoded by the Gag gene [12]. Each CA monomer consists of two independently folded domains, the N-terminal domain (NTD) and the C-terminal domain (CTD), connected by a flexible linker (Figure 1) [13,14]. The NTD contains seven alpha helices and one beta-hairpin, while the CTD comprises four alpha helices [15]. In function, the NTD primarily mediates the formation of hexameric and pentameric structures, whereas the CTD is predominantly involved in interconnections between these multimers [15,16].

The mature capsid comprises approximately 1500 CA monomers, forming around 250 hexamers and 12 pentamers [13,17]. The inclusion of pentamers enables the structural closure of the capsid. The hexamers are formed through the tight cohesion of adjacent CA monomers via NTD-NTD interactions along the six-fold axis and NTD-CTD interactions. Adjacent hexamers then interact via homologous CTD-CTD dimer interfaces at two-fold and three-fold symmetry axes, forming a hexagonal lattice [14,18]. This lattice achieves closure by incorporating 12 CA pentamers. Moreover, these pentamers are primarily localized in high-curvature regions such as the conical apex and base. The hexameric and pentameric capsid proteins work synergistically to achieve the construction of a perfect fullerene conical capsid. When perfecting, the resulting core is tightly closed, preventing cellular components from accessing the genetic material [19,20]. Compared to hexamer, the pentamer exhibit a ~19° rotation of the NTD around the five-fold axis and altered spatial relationships between the NTD and CTD. While the CTD dimer interface between pentamers and adjacent hexamers resembles that of hexamer–hexamer interactions, the helical arrangement at three-fold interfaces near pentamers is more compact to accommodate the higher curvature. Recent studies [21,22] reveal that IP6 (inositol hexakisphosphate) binding stabilizes pentamer conformations. Moreover, the relative motion between the NTD and CTD of CA monomers, as well as the subtle flexibility in CA-CTD interactions at the two-fold and three-fold interfaces of hexamers within the mature capsid, provides the necessary twist and tilt to accommodate the variable curvature of the conical structure [20].

In recent years, significant progress has been made in the research on HIV-1 capsid inhibitors. In this review, we summarize the latest research achievements in this field, with a particular focus on the molecular mechanisms by which small-molecule compounds modulate the structural stability and assembly–disassembly processes of the capsid. By gaining an in-depth understanding of the interaction mechanisms between small-molecule drugs and the viral capsid, this will not only help uncover new therapeutic targets, but will also provide crucial theoretical foundations and innovative strategies for developing novel anti-HIV-1 drugs with enhanced efficacy and improved safety.

## 2. New Advances Regarding the Structural Aspects of Novel HIV-1 Capsid Protein

Notably, the HIV-1 capsid is a metastable structure, and ensuring assembly and disassembly of the capsid occur at the right time and in the right environment can precisely regulate the process of HIV-1 replication [23,24]. During the early stage of HIV-1 infection, capsid uncoating releases the viral genome into the host cell, enabling replication and infection [25,26]. Both premature and delayed uncoating impair viral infectivity: the host factor TRIM5α recognizes the HIV-1 capsid and accelerates uncoating to suppress HIV-1 reverse transcription, while an overly stable capsid hinders efficient genome release into the nucleus. Currently, there is ongoing debate regarding the timing and mechanism of viral capsid uncoating [9]. One hypothesis suggests that uncoating occurs during capsid transport through the cytoplasm [27], while another proposes that capsids disassemble after reaching and docking with nuclear pore complexes [28]. Additionally, some studies [29,30,31,32] indicate that intact or partially uncoated capsids are imported into the nucleus, where complete disassembly follows reverse transcription. Recent research [33] has further supported the newly proposed intra-nuclear uncoating model, demonstrating that HIV-1 capsids undergo a gradual dissociation process after nuclear entry. This process involves the initial formation of localized defects in the capsid lattice, followed by the progressive loss of CA proteins, and ultimately culminating in complete capsid disintegration. Moreover, in the late replication phase, CA proteins are essential for virion assembly and maturation, as only structurally intact viral particles retain infectivity [25,26]. Therefore, interfering with or disrupting the oligomerization process of the capsid protein can hinder the proper assembly of the viral particles. Alternatively, inducing abnormal disassembly of the capsid, that is, disrupting any step of the capsid assembly process, will potentially inhibit the replication of HIV-1. This underscores HIV-1 CA as an ideal therapeutic target. The recent FDA approval of **lenacapavir**, the first-in-class capsid inhibitor for HIV-1 treatment [34], validates the feasibility of capsid-targeted antiviral strategies and opens new avenues for developing novel therapeutics against viral pathogens.

In recent years, breakthroughs in the structural biology techniques of cryo-electron microscopy (cryo-EM) and computational modeling (AlphaFold) have provided profound insights into the assembly mechanisms of the HIV-1 capsid, enabling researchers to resolve the three-dimensional conformations of CA and its assembly dynamics at near-atomic resolution [22]. By integrating cryo-EM with single-particle analysis, Dick and colleagues [21] determined the structural continuity of hexameric and pentameric lattices within the capsid. Furthermore, Yang et al. [35] used ^19^F NMR spectroscopy and extended-timescale molecular dynamics (MD) simulations to investigate the dynamic conformational changes in the CA-CTD dimers, successfully characterizing a previously elusive “cryptic” D2 dimeric state and providing a novel perspective on the dimeric states of HIV-1 CA. These technological advances have significantly deepened the understanding of capsid architecture and assembly dynamics, paving the way for CA-targeted antiviral strategies. By focusing on the HIV-1 CA protein, such strategies hold promise to overcome the limitations of conventional therapies, potentially enabling the development of a next generation of antiviral agents with broad-spectrum activity, high resistance barriers, and reduced toxicity.

## 3. Small Molecular Inhibitors for HIV-1 Capsid

Small-molecule inhibitors are a class of low-molecular-weight compounds (typically MW < 1000 Da) characterized by relatively simple chemical synthesis, high structural tunability, good cell permeability, and the ability to specifically target critical sites on viral capsid proteins. Additionally, owing to the high conservation of the HIV-1 CA gene sequence, small-molecule drugs targeting CA exhibit exceptional resistance profiles and therapeutic durability. By designing small molecules to bind distinct functional sites on CA, these compounds disrupt protein–protein interactions (PPIs), destabilize the capsid structure, and ultimately impede proper capsid assembly or normal uncoating, thereby effectively suppressing HIV-1 infectivity. Over decades of research, numerous small-molecule inhibitors targeting HIV-1 CA have been developed and evaluated for their anti-HIV-1 activity. In 2021, Sun et al. [36] provided a detailed review of the last developments in HIV-1 capsid-targeting inhibitors. We will summarize the latest advances in this field since 2020 and focus on the molecular interactions of the novel compounds (Table 1) with the HVI-1 capsid.

### 3.1. ***PF74*** and Its Derivatives

**Figure 2:**
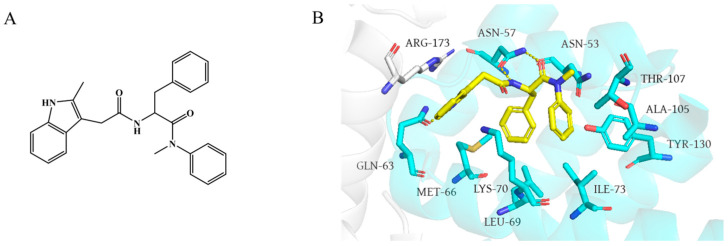
(**A**) Chemical structures of **PF74**. (**B**) Binding modes of **PF74** to the NTD-CTD interface of CA (PDB: 5HGL). Yellow dashed lines indicate hydrogen-bond interactions.

First, it is worth noting the small-molecule inhibitors represented by **PF74**, **lenacapavir**, and their derivatives. Currently, the most extensively studied HIV-1 capsid inhibitor is **PF-3450074** (abbreviated as **PF74**), which was developed by Pfizer through structural optimization based on the high-throughput screening hit **PF-1385801**. **PF74** exhibits broad inhibitory activity against HIV isolates and functions at both the early and late stages of viral replication and infection. It is a phenylalanine-derived peptidomimetic compound that consists of a phenylalanine core, an aniline moiety, and a methylindole ring (Figure 2A). It exerts its antiviral effects by binding to the interface between the NTD and CTD of HIV-1 CA. As illustrated in Figure 2B, the phenylalanine backbone primarily interacts with residues such as Asn-57, Lys-70, Met-66, and Leu-69, while the aniline group engages Asn-53, Thr-107, Ala-105, and Tyr-130. The methylindole ring stacks with Arg-173 in the adjacent CTD via cation–π interactions and forms a hydrogen bond with Gln-63 in the NTD [18,37]. Intriguingly, **PF74** exhibits a concentration-dependent bimodal mechanism [38,39]. At low concentrations (below 2 nM), it competes with host factors CPSF6 and NUP153 for CA binding, thereby impairing nuclear entry. At higher concentrations, **PF74** disrupts inter-hexameric CA interactions, inducing premature capsid uncoating and blocking reverse transcription during the early stages of the viral life cycle. Additionally, in the late stage of HIV-1 replication, **PF74** accelerates capsid protein assembly, interfering with the formation of morphologically normal viral particles and inhibiting viral maturation. Despite its potent antiviral phenotype, **PF74** suffers from suboptimal metabolic stability and limited antiviral potency [48], driving extensive efforts to optimize its structure through derivative design to enhance its pharmacological properties [49,50,51,52,53,54].

**Figure 3:**
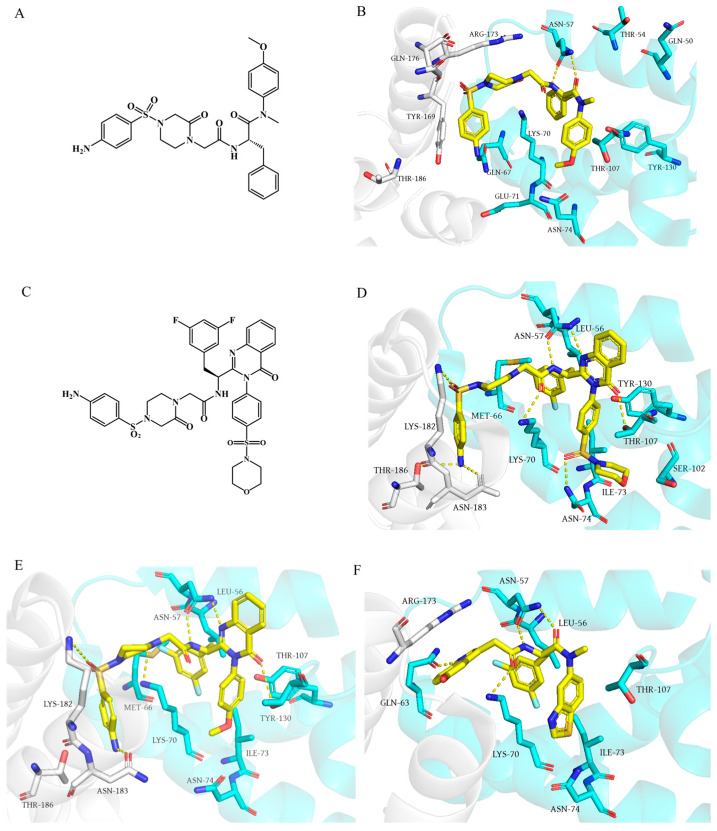
Chemical structures of (**A**) **11L** and (**C**) **21a2**. Binding modes of several representative inhibitors to the interface of NTD-CTD: (**B**) **11L** (PDB: 8F22); (**D**) **21a2** (PDB: 8TOV); (**E**) **12a2** (PDB: 8TQP); (**F**) **IC-1k** (PDB: 8V17). Yellow dashed lines indicate hydrogen-bond interactions.

In 2020, Sun et al. [55] enhanced both the antiviral activity and metabolic stability of **PF74** by replacing the indole moiety with substituted benzenesulfonamide groups. Among these derivatives, compound **11L** (Figure 3A) emerged as the most potent, exhibiting an *EC*_50_ value of 90 nM in HIV-1 NL4-3 infected TZM-bl cells, with 5.8-fold higher activity than **PF74**. Structure–activity relationship (SAR) studies revealed that introducing a piperazinone moiety significantly improved antiviral efficacy. Surface plasmon resonance (SPR) assays confirmed the direct binding of **11L** to HIV-1 CA, with preferential affinity for CA monomers. MD simulations further demonstrated that the benzene ring in **11L**’s core interacts with Lys70, while its methoxy group forms a hydrogen bond with Asn74. Additionally, the oxopiperazine α-proton at the substituent region engages Asn57. Compared to **PF74**, the newly introduced aminobenzenesulfonyl group in **11L** establishes additional hydrogen bonds with Thr54, Gly106, and Gln50, markedly strengthening interactions with HIV-1 CA monomers (Figure 3B). This binding likely locks CA monomers into an open conformation, potentially rigidifying hexamers into a uniform state and disrupting their functional dynamics, and thereby accelerating early-stage capsid disassembly. Moreover, the p24 content analysis and in vitro capsid assembly assays performed in the same study suggest that **11L** may also impair viral particle maturation at later stages, generating non-infectious virions. Further experiments need to be conducted in follow-up research.

Subsequent modifications of compound **11L** with 4-sulfonyl morpholine and 4-sulfonyl thiomorpholine-1,1-dioxide groups performed by Xu et al. [40] yielded novel HIV capsid modulators **12a2** and **21a2** (Figure 3C), which exhibited 2.5-fold and 7.3-fold higher anti-HIV-1 activity compared to the **11L** and **PF74**, respectively. SPR assays and X-ray co-crystal structures revealed that **12a2** and **21a2** specifically bind to the interface between the CTD and NTD of HIV-1 CA hexamers, occupying multiple pockets and forming extensive interactions to stabilize binding across CA regions, thereby disrupting normal CA-CA interactions. As illustrated in Figure 3D,E, the compounds establish intricate hydrogen-bond networks with residues Thr107, Asn57, Lys70, Lys182, and Thr183. The 3,5-difluorophenyl substituent embeds into a hydrophobic pocket formed by Leu56, Val59, Met66, Leu69, Lys70, and Ile73. Additionally, **21a2** forms extra hydrogen bonds with Asn186 and Asn74. Similarly to PF74, **12a2** and **21a2** inhibit both early and late stages of HIV-1 replication. Moreover, FRET-based assays demonstrated that 50 μM **12a2** and **21a2** significantly accelerate CA assembly, while ELISA confirmed that p24 levels were unchanged but infectivity was reduced, suggesting the generation of unstable, malformed capsids.

Additionally, by modifying the aniline group and indole ring of **PF74**, Xu et al. [56] discovered a potent benzothiazole-containing compound **7u**, which exhibits dual-phase inhibitory properties. This compound interacts with the CA hexamer, occupying critical regions of the NTD-CTD interface, and inhibits viral replication during both early and late stages of infection. Similarly to **PF74**, **7u** demonstrates stronger inhibitory efficacy in the early stage of HIV-1 replication compared to the late stage. The study hypothesizes that **7u** may stabilize the CA hexamer and disrupt capsid disassembly. Furthermore, **7u** shows slightly improved metabolic stability in human liver microsomes (HLM) compared to the **PF74**, providing hope that the metabolic stability of the inhibitor will be further improved.

Later on, the same team identified a potent HIV-1 CA modulator, **IC-1k** (*EC*_50_ = 2.69 nM), which demonstrates markedly enhanced metabolic stability in HLM, with a half-life (T_1/2_ = 91.3 min) that is 130-fold longer than that of the **PF74** [41]. **IC-1k** preferentially binds to CA hexamers, adopting a structural orientation similar to compounds **12a2** and **21a2**. Its 3,5-difluorinated phenyl ring embeds within a hydrophobic pocket formed by Leu56, Met66, Leu69, Lys70, and Ile73, while forming extensive hydrogen-bond interactions with residues Asn57, Gln63, Lys70, and Asn74. The benzothiazole moiety occupies a pocket defined by Thr107 and Lys70, acting as a rigid scaffold that restricts capsid conformational flexibility (Figure 3F). This forced loss of CA plasticity underpins the high antiviral efficacy of **IC-1k**.

Of note, although **compounds 24** [57] and **6a-9** [42] were reported early in 2020, their molecular modes of function have not been explored before; we therefore incorporated a related structural analysis into this review. Wang et al. [57] designed and synthesized a series of novel small-molecule compounds by modifying the indole ring of PF74 to improve its metabolic stability. Among them, the 2-indolone sub-chemotype 24 significantly decreased the melting temperature of the CA hexamer (Δ*Tm* = −2.4 °C). Further studies revealed that, unlike **PF74**, the 3-chloroaniline group of **compound 24** formed a halogen bond with the CA residue N53. Therefore, it directly destabilized the CA hexamer. Moreover, Sun et al. [42] designed a series of 4-phenyl-1H-1,2,3-triazole phenylalanine derivatives through scaffold-hopping and click chemistry. One of these derivatives, **6a-9** (*EC*_50_ = 3.13 μM), exhibited significant antiviral activity and slightly improved metabolic stability both in HLM and human plasma compared with **PF74**. Further SPR assays indicated that it had higher affinity for the CA hexamer than for the CA monomer. Additionally, it was more effective in the late stages of the HIV replication cycle than in the early stages, making it comparable to **PF74**. Moreover, the molecular dynamics simulations showed that **6a-9** formed hydrophobic interactions and hydrogen bonds with residues such as Lys70, Leu56, Ile73, Ala105, Met66, Asn74, and Thr107. Further quantitative p24 assays demonstrated that the number of virus particles produced in the presence of **6a-9** was only slightly reduced (by about 15%), and in vitro capsid assembly experiments indicated that **6a-9** neither accelerated nor decreased CA assembly. Therefore, it was speculated that compound **6a-9** might bind to the assembled capsid protein, disrupting the normal morphology of the mature capsid in the virus and preventing the virus particles from forming properly, thereby inhibiting late-stage viral infection.

### 3.2. ***Lenacapavir*** and Its Derivatives

**Figure 4:**
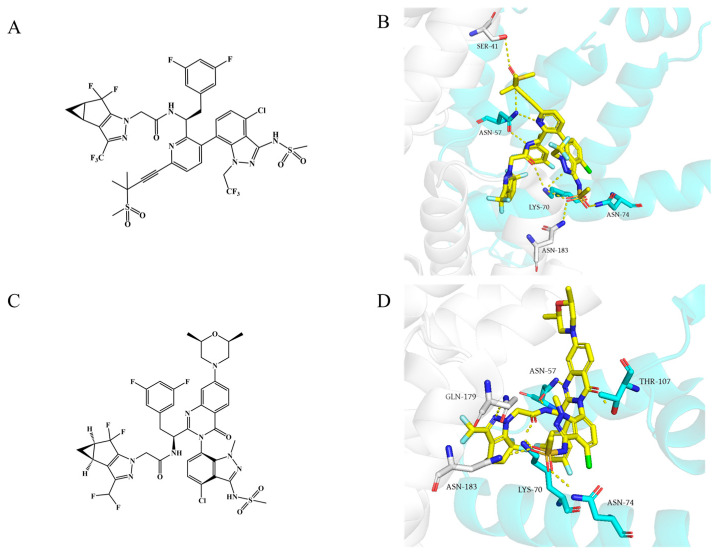
Chemical structures of (**A**) **Lenacapavir** and (**C**) **GSK878**. Binding modes of (**B**) **Lenacapavir** (PDB: 6V2F) and (**D**) **GSK878** (PDB: 8FIU) to NTD-CTD. Yellow dashed lines indicate hydrogen-bond interactions.

Gilead Sciences modified the **PF74** scaffold by incorporating multiple hydrogen bond donors and acceptors to enhance its affinity for the HIV-1 CA, resulting in the development of the famous **lenacapavir**, also known as **GS-6207**, with the trade name of Sunlenca (Figure 4A). As the first-in-class, long-acting, ultra-potent HIV-1 CA inhibitor, **lenacapavir** demonstrated remarkable potency, with an *EC*_50_ of 105 pM in the HIV-1-infected MT-4 cells, and *EC*_50_ values of 32 pM and 56 pM against HIV-1 in primary human CD4 T cells and macrophages, respectively [43]. It exhibits high metabolic stability and exceptional antiviral efficacy. **Lenacapavir** received approval from the U. S. Food and Drug Administration (FDA) on 22 December 2022, for the treatment of multidrug-resistant HIV-1 infection [34].

**Lenacapavir** binds to the same pocket as **PF74** at the CA interface, but works in an opposite way. Unlike **PF74**, which destabilizes the capsid lattice and accelerates capsid disassembly, **lenacapavir** stabilizes the capsid lattice and impedes uncoating [44,58]. It tightly interacts with a hydrophobic pocket formed by two adjacent CA monomers, exhibiting shape complementarity. This binding rigidifies both intra- and inter-hexamer interactions, thereby stabilizing the capsid [59]. Additionally, **lenacapavir** forms extensive electrostatic, hydrophobic, and hydrogen-bond interactions with the CA1-NTD, CA2-CTD, and CA2-NTD regions (Figure 4B). Specifically, it establishes a complex hydrogen-bond network with Asn57, Lys70, and Asn74 in CA1-NTD, Ser41 in CA2-NTD, and Gln179 and Asn183 in CA2-CTD [43,59]. Compared to the binding with the CA monomer, **lenacapavir** exhibits higher affinity for the CA hexamer (*K_D_* is approximately 200 pM). Each **lenacapavir** molecule binds between adjacent CA subunits, stabilizing CA dimers, inducing an open CA conformation, and restricting CA flexibility. This promotes the formation of highly stable hexamers while specifically blocking pentamer formation [60], ultimately leading to disruption of the conical apex in the structure and the generation of aberrant capsids [44]. Furthermore, **lenacapavir** alters the stability of Gag/Gag-Pol complex, reducing viral assembly and release [43]. Therefore, **lenacapavir** inhibits viral replication by blocking nuclear entry, disrupting the capsid disassembly process, impairing the assembly of mature capsids, and affecting their release.

Of note, clinical trials have demonstrated its efficacy in multidrug-resistant HIV-1 patients, with a dosing regimen of subcutaneous administration every six months, highlighting its potential as a long-acting therapeutic option [61,62]. Currently, **lenacapavir** is undergoing Phase III PURPOSE 1 trials for PrEP [63,64]. Interim results show 100% efficacy in protecting participants from HIV-1 infection via subcutaneous injection. Additionally, feasibility studies are exploring the annual intramuscular administration of **lenacapavir** to further enhance its clinical utility [65].

However, a recent study shows that the emergence of HIV-1 CA drug-resistant mutants has affected the binding of **lenacapavir**, resulting in a significant reduction in its antiviral activity [66]. These mutants include L56I, M66I, Q67H, K70N, N74D/S, Q67H/T107N, and Q67H/N74S [43]. Such mutations reduce the antiviral activity of **lenacapavir** by factors ranging from 6 to 3200. For example, the Q67H mutation induces conformational changes in the binding pocket, reducing the binding affinity of **lenacapavir** for the CA hexamer. The N74D mutation eliminates the hydrogen bond between the original Asn74 and the sulfonamide group of **lenacapavir**. Moreover, after the N74D mutation, Asn74 is replaced by negatively charged Asp74, which generates electrostatic repulsion with the sulfonamide group of **lenacapavir**, further weakening the binding. Especially, the double mutation of Q67H/N74D further exacerbates the binding obstacle of **lenacapavir** [67]. Additionally, the M66I mutation replaces Met66 with Ile66, whose β-branched side chain causes spatial conflicts with the difluorobenzyl and cyclopentylpyrazole groups of **lenacapavir**, preventing stable binding [68]. Therefore, it is necessary to design new subtypes to inhibit the development of drug resistance.

Kvaratskhelia and colleagues [67] replaced the cyclopentylpyrazole ring of **lenacapavir** with a tetrahydroindazole ring and removed the cyclopropyl and fluorine atoms, resulting in the compound **KFA-012**. Compared with **lenacapavir**, **KFA-012** (*EC*_50_ = 9.177 nM) exhibited approximately 2.6-fold higher antiviral activity against the Q67H/N74D mutant of HIV-1. The flexibility of the cyclohexene ring allows it to better accommodate the conformational changes in His67, reducing steric hindrance and thereby enhancing its antiviral activity. Additionally, Akther et al. [69] reported work on the design of **GS-6207** subtypes: although the antiviral effects were not satisfactory as expected, this provided ideas for the design and synthesis of new subtypes in future to better overcome existing drug-resistance barriers.

It is worth noting that a recently discovered, highly effective HIV-1 CA inhibitor, **GSK878** (Figure 4C), occupies the same pocket as **PF74** and **lenacapavir** [45]. This is a novel HIV-1 capsid inhibitor based on a quinazolin-4-one scaffold, with higher antiviral activity (*EC*_50_ = 39 pM); however, unfortunately, M66I, L56I, and Q67H/N74D still significantly reduce the sensitivity of HIV-1 to **GSK878** [46]. As illustrated in Figure 4D, it forms hydrogen bonds with Thr107, Asn57, Asn74, Lys70, and Asn183, while the 3,5-difluorophenyl group is embedded in the hydrophobic pocket composed of Leu56, Ile73, Met66, and Tyr130 and the indole substituent engages in a cation-π interaction with Lys70 [45]. Unlike **lenacapavir**, the carbonyl group in the newly introduced 4-quinazolinone ring of **GSK878** forms a new hydrogen bond with Thr107. **GSK878** has a dual-stage mechanism of action; but its antiviral potency mainly stems from early inhibition; that is, it disrupts nuclear entry and alters the stability of the CA core [46]. Moreover, in fate-of-the-capsid assays, there was a clear increase in pelletable CA; however, the p24 release was significantly reduced, indicating that **GSK878** inhibits viral genome release and viral particle production by enhancing the stability of the CA hexamer. Such processes prevent the CA core from disassembling at the right time and place, thereby blocking viral replication. Although **GSK878** was not advanced due to significant liver damage, its development provided key structural and mechanistic insights for the subsequent development of HIV-1 novel capsid inhibitors using the quinazolin-4 as a scaffold. Akther et al. [70] designed a series of **GSK878** derivatives based on the core structure of **GSK878**. These compounds exert antiviral effects by binding to the NTD-CTD interface of HIV-1 CA, although their antiviral activity and drug resistance still need further optimization.

### 3.3. Other Molecules

In addition to extensively studied small-molecule drugs such as **PF74** and **lenacapavir**, other novel small-molecule inhibitors have also been developed in recent years. Small-molecule inhibitors like **ACAi-028** [47] with novel target sites in hydrophobic pockets on HIV-1 capsids have been developed. These advancements provide diverse compounds and targeted strategies for anti-HIV-1 drug research and development. Moreover, new antiviral agents could offer alternative treatment options for drug-resistant patients, while the future development of novel capsid inhibitors may address the current limitations in their broad-spectrum antiviral activity.

Kobayakawa et al. [71] identified a novel HIV-1 capsid inhibitor, **MKN-1**, through computer-aided drug design. **MKN-1A** exhibited significant anti-HIV-1 activity (*EC*_50_ = 8.0 µM), while its diastereomer **MKN-1B** showed significantly reduced activity (*EC*_50_ = 24 µM), confirming that the compound acts through stereospecific binding to the CA target. Experiments showed that **MKN-1** significantly inhibited the production of the viral protein. Molecular docking studies provided a detailed indication that the compound targets the Trp184/Met185 interface region of the CA monomer, disrupting the hydrophobic interactions between these two residues and thereby interfering with CA protein oligomerization through reducing the formation of infectious viral particles, and effectively inhibiting HIV-1 replication.

In follow-up studies, the team developed **MKN-3** and its derivatives, including **TKB063**, **TON02**, and **TON03**, via computer-aided design [72]. Among them, TKB063 exhibited superior anti-HIV activity (*EC*_50_ = 4.8 µM) and low cytotoxicity (CC_50_ > 50 µM), highlighting its potential as a novel anti-HIV therapeutic. Similarly to **MKN-1**, **TKB063** targets the Trp184-Met185 site of the CA protein, blocking hydrophobic interactions to destabilize the CA oligomer. This disruption leads to abnormal viral particle morphology and loss of infectivity. Notably, the introduction of an isopropyl group to the secondary amine nitrogen of the homopiperazine ring in **TKB063** enhanced its hydrophobicity (logP = 1.31), improving cell membrane permeability and thereby boosting antiviral efficacy. Recent studies showed that replacing the methylthio group in **MKN-1** with a methylene group yielded **MKU-010** [73], which slightly improved anti-HIV activity. SAR analysis suggests that moderate hydrophobicity and smaller substituents favor binding to the CA protein. Future research may focus on the structural optimization of **MKN-1** derivatives to further explore SAR and develop more potent and specific anti-HIV agents.

**ACAi-028** [47] specifically targets a novel hydrophobic pocket in the HIV-1 CA-NTD and primarily acts during the early stages of HIV-1 replication, without affecting late-stage processes. **ACAi-028** forms hydrogen-bond interactions with Gln13, Ser16, and Thr19 residues in the CA-NTD, tightly binding to the hydrophobic pocket. By binding to CA, **ACAi-028** significantly reduces the thermal stability of CA, inhibits CA polymerization, disrupts HIV-1 capsid disassembly, and consequently blocks early HIV-1 replication. Therefore, its novel targeting site on the CA-NTD provides a new direction for developing long-acting and broad-spectrum inhibitors. Previous studies have indicated that heme and protoporphyrin exhibit inhibitory effects against HIV-1 infection [74]. Recently, Zhang et al. [75] confirmed that **protoporphyrin IX** (**PPIX**) can act as a modulator of HIV-1 CA assembly. **PPIX** exhibits significantly higher binding affinity to CA hexamers (*K_D_* = 0.53 μM) compared to monomers (*K_D_* = 4.30 μM). Further molecular docking and dynamics simulations revealed that **PPIX** binds tightly to the NTD-CTD subunit interface of CA hexamers, forming hydrogen bonds with L211 and E212 residues in the CA-CTD and the T72 residue in the CA-NTD. Acting like a “molecular glue”, **PPIX** strengthens interactions between CA subunits, accelerating capsid polymerization and promoting the formation of abnormal capsid structures, thereby inhibiting viral replication. However, the current research is limited to in vitro studies, and further validation of its in vivo antiviral activity, toxicity, and effects on mature viral particles is required.

In addition, the natural compounds **sennoside A** (**SA**) and **sennoside B** (**SB**) have been demonstrated to exhibit anti-HIV-1 activity and are potential modulators of capsid assembly [76]. In 2.5 M NaCl conditions, **SA** and **SB** promote in vitro HIV-1 CA assembly in a dose-dependent manner, with dissociation constants (*K_D_*) of 1.75 μM and 2.25 μM for the binding to the CA hexamer, respectively. Both compounds tightly bind to the CA hexamer, accelerating the CA assembly process and promoting capsid polymerization. Molecular docking results further indicate that they bind to the NTD-CTD inter-subunit interface formed between two adjacent monomers within the CA hexamer. **SA** forms a hydrogen-bond network with residues N57, V59, Q63, K70, and N74 of the CA1-NTD and residue Q179 in CA2-CTD, while SB establishes hydrogen-bond interactions with residues N53, K70, and N74 in CA1-NTD, and residues A177 and Q179 of the CA2-CTD. However, it remains unclear whether **SA** and **SB** are effective in vivo capsid inhibitors; this needs to be further validated.

Recently, Artía et al. [77] utilized an AI platform based on a deep neural network (AtomNet^®^) to conduct a large-scale screening targeting the conserved NTD region of HIV-1 CA, adjacent to the Nup153 binding site. This effort identified 84 potential HIV-1 capsid assembly-disrupting molecules. By measuring absorbance changes in recombinant HIV-1 capsid proteins (CANC proteins), compounds **C6**, **C19**, **C44**, **C61**, **C72**, and **C78** were found to accelerate CANC polymerization by over 40% compared to the control reaction. This suggests that these compounds may disrupt normal viral assembly by accelerating capsid protein polymerization, potentially leading to abnormal capsid structures. MST experiments performed in the same study revealed that **C37** and **C29** exhibited 2-fold and 5-fold higher binding affinity, respectively, for the drug-resistant mutant CA protein (Q67H/N74D) compared to the wild type, partially overcoming viral resistance and demonstrating potential antiviral activity. Further molecular dynamics simulations showed that **C37** engages in π-stacking interactions with His67 and enhances interactions with Lys70, while **C29** forms a novel interaction network with residues Leu56, Asn57, Gln63, Leu69, Lys70, and Ile73, maintaining high affinity and thereby inhibiting the replication of drug-resistant viral strains.

Additionally, the novel HIV-1 capsid inhibitors **VH4004280** [78] and **VH4011499** [79] have recently entered clinical trials, expanding therapeutic and preventive options for HIV-1 while providing critical momentum for the development of next-generation anti-HIV drugs.

## 4. Conclusions and Perspectives

HIV-1 CA plays a critical role in the viral life cycle and is highly conserved, making it an attractive therapeutic target for novel antiviral drugs. Extensive research has been conducted on HIV-1 capsid inhibitors, which can either accelerate or inhibit capsid assembly/disassembly, thereby exerting antiviral effects. These processes are summarized in this review, which essentially highlights the recent advancements in the field and elucidates the assembly mechanisms of the HIV-1 capsid protein and its interactions with small-molecule drugs. It is hoped that this review will further enhance the understanding of HIV-1 inhibition mechanisms, facilitating the future exploration of the development of novel capsid inhibitors.

Notably, the successful approval of **lenacapavir** and its remarkable efficacy in preventing HIV-1 infection have brought hope that the HIV epidemic will be ended [80]. This achievement further confirms that targeting capsid assembly is an effective and highly promising strategy against viral infections. Additionally, advancements in structural biology technologies have deepened our understanding of viral capsid assembly mechanisms. The discovery of potential action modes of capsid proteins may provide new therapeutic targets and innovative approaches for designing next-generation HIV-1 capsid inhibitors.

However, the field still faces many challenges. These primarily include the antiviral efficiency of capsid inhibitors, the interaction mechanisms between small-molecule drugs and the HIV-1 capsid, drug resistance caused by viral mutations, and discrepancies between in vitro models and the in vivo environment, along with associated adverse effects. These factors significantly limit the translational potential and clinical applications of capsid inhibitors. Of course, combination therapies utilizing inhibitors with different mechanisms of action or multi-target agents may enhance overall antiviral activity. For example, combining capsid inhibitors with other antiviral drugs, such as protease inhibitors or integrase inhibitors, could improve therapeutic efficacy while reducing the risk of drug resistance [81,82,83]. Furthermore, the integration of artificial intelligence (AI) technologies has greatly facilitated drug development. AI-driven drug design approaches enable the rapid screening of compounds with potential antiviral activity, followed by subsequent optimization and synthesis [84], offering promising avenues for overcoming existing challenges.

In conclusion, continued in-depth research into the assembly processes of the HIV-1 capsid and the interaction mechanisms between small-molecule drugs and the capsid is critical for developing effective antiviral interventions in the future. Additionally, future studies must prioritize strengthening translational efforts from investigations of antiviral efficacy and fundamental inhibitory mechanisms to clinical trials, with the goal of developing safe, effective anti-HIV-1 therapies with minimal side effects.

## Figures and Tables

**Table 1:** Representative compounds targeting HIV-1 CA.

Compounds	Structure	*EC*_50_	Life Cycle Stage	Ref.
**PF74**	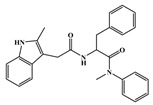	0.61 μM	early/late	[37,38,39]
**21a2**	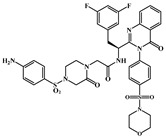	0.11 μM	early/late	[40]
**IC-1k**	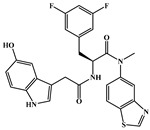	2.69 nM	early/late	[41]
**6a-9**	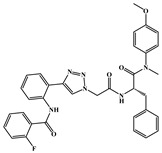	3.13 μM	early/late	[42]
**Lenacapavir**	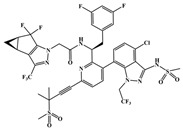	32 pM	early/late	[43,44]
**GSK878**	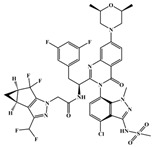	39 pM	early/late	[45,46]
**ACAi-028**	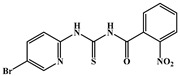	0.12 μM	early	[47]

## Data Availability

Data are contained within the article.
